# Continuous ECS-indicated recording of the proton-motive charge flux in leaves

**DOI:** 10.1007/s11120-013-9884-4

**Published:** 2013-07-17

**Authors:** Christof Klughammer, Katharina Siebke, Ulrich Schreiber

**Affiliations:** 1 Julius-von-Sachs Institut für Biowissenschaften Universität Würzburg, Julius-von-Sachs Platz 2, 97082 Würzburg, Germany; 2 Heinz Walz GmbH, Eichenring 6, 91090 Effeltrich, Germany

**Keywords:** CO_2_ gas exchange, DIRK method, Dual-PAM-100, Electrochromic absorbance shift, Photosynthetic electron transport, P515

## Abstract

Technical features and examples of application of a special emitter–detector module for highly sensitive measurements of the electrochromic pigment absorbance shift (ECS) via dual-wavelength (550–520 nm) transmittance changes (P515) are described. This device, which has been introduced as an accessory of the standard, commercially available Dual-PAM-100 measuring system, not only allows steady-state assessment of the proton motive force (pmf) and its partitioning into ΔpH and Δ*Ψ* components, but also *continuous* recording of the overall charge flux driven by photosynthetic light reactions. The new approach employs a double-modulation technique to derive a *continuous* signal from the light/dark modulation amplitude of the P515 signal. This new, continuously measured signal primarily reflects the rate of proton efflux via the ATP synthase, which under quasi-stationary conditions corresponds to the overall rate of proton influx driven by coupled electron transport. Simultaneous measurements of charge flux and CO_2_ uptake as a function of light intensity indicated a close to linear relationship in the light-limited range. A linear relationship between these two signals was also found for different internal CO_2_ concentrations, except for very low CO_2_, where the rate of charge flux distinctly exceeded the rate of CO_2_ uptake. Parallel oscillations in CO_2_ uptake and charge flux were induced by high CO_2_ and O_2_. The new device may contribute to the elucidation of complex regulatory mechanisms in intact leaves.

## Introduction

Progress in photosynthesis research has been driven to a large extent by the development of new measuring techniques and methodology. Outstanding examples are Pierre Joliot’s pioneering developments in amperometric techniques for oxygen detection (Joliot 1956, 1968) and in absorption spectrophotometry (Joliot et al. 1980, 2004), which have led to numerous important discoveries and have been stimulating generations of photosynthesis researchers. Our present contribution describes a new instrument for continuous measurements of the electrochromic absorbance shift in vivo, i.e., a topic that has been close to the heart of Pierre Joliot for at least 40 years. We dedicate this paper to him and to Govindjee on the occasion of their 80th birthdays.

During the past 50 years the major mechanisms involved in the complex process of photosynthesis have been elucidated by basic research using isolated chloroplasts or membrane fragments (with substantial contributions by both Pierre Joliot and Govindjee). Some important open questions have remained, in particular regarding the regulation of the highly complex in vivo process in response to environmental factors, which limit the rate of CO_2_-assimilation and consequently plant growth. Obtaining reliable information on the intact system, as close as possible in its natural state, is complicated not only by the much higher degree of complexity, but also by various aggravating factors affecting the quality of optical probes. While measurements of the overall rate of CO_2_-uptake or O_2_-evolution in intact leaves are relatively simple and straightforward, specific absorbance changes due to various electron transfer steps are covered by much larger broadband absorbance changes due to electrochromic pigment absorbance shifts and light scattering changes. Furthermore, leaf transmittance in the visible spectral region is low due to high Chl content and the strongly increased path length of measuring light (ML) by multiple scattering. Another complicating factor is the need to keep the time-integrated intensity of the ML to a minimum, so that its actinic effect does not change the state of the sample. Therefore, in vivo optical spectroscopy in the visible range is a challenging task.

Large broadband absorbance changes are observed upon continuous illumination of intact leaves peaking at 505, 515–520, and 535 nm, all of which are closely related to the proton motive force (pmf) generated by proton coupled electron transport. The absorbance increase at 505 nm reflects formation of zeaxanthin via de-epoxidation of violaxanthin induced upon acidification of the thylakoid lumen (Yamamoto et al. 1972; Bilger et al. 1989). Zeaxanthin changes are slow and can be kinetically differentiated from faster 515–520 nm and 535 nm changes. The absorbance increase peaking at 515–520 nm is caused by an electrochromic shift of absorption of various photosynthetic pigments, including carotenoids (Junge and Witt 1968). It has been described by the abbreviated terms P515, carotenoid shift or ECS. In the present communication, the terms ECS and P515 are used interchangeably. The ECS (P515) signal may be considered an intrinsic optical voltmeter that rapidly responds to changes of the electrical potential across the thylakoid membrane (Witt 1971, 1979; Joliot and Joliot 1989). Photosynthetic electron transport involves three electrogenic reactions, namely the two photoreactions (PS I and PS II) (Witt 1971) and the Q-cycle of the cyt bf complex (Velthuys 1978; Joliot and Joliot 1986). While the ECS due to PS I and PS II responds without measurable delay to the onset of light, the ECS caused by the Q-cycle responds with a time constant in the order of 10 ms to light. Finally, the absorbance increase around 535 nm for long has been attributed to a light induced increase of light scattering caused by internal acidification of the thylakoids (Heber 1969). It has been used in numerous in vivo studies as a convenient semi-quantitative optical probe of “membrane energization” and of the ΔpH component of the pmf in intact leaves. It closely correlates with the fluorescence-based indicators of “energization” q_E_ and NPQ (see e.g., Bilger et al. 1988). While it has been assumed that 535 nm changes are caused by changes in grana stacking, this interpretation recently has been questioned by Ruban et al. (2002) who suggest that the 535 nm increase of absorbance is due to a red shift of the zeaxanthin absorption peak. Therefore, when the 535 nm changes are referred to as “light scattering” changes, this is done with quotation marks.

The original Joliot-type kinetic spectrophotometer (Joliot and Delosme 1974; Joliot et al. 1980) was developed for highly sensitive measurements of flash relaxation kinetics in suspensions of algae and thylakoid membranes (i.e., for conditions avoiding the complications resulting from overlapping 535 and 505 nm changes that are characterized by relatively slow kinetics during continuous illumination). Absorption was measured during each of a series of 2 μs monochromatic flashes given at various intervals after the actinic flashes (pump-and-probe method). While the intensity of individual probe flashes was much higher than that of continuous ML in conventional devices, thus resulting in a correspondingly high signal/noise ratio, the integrated actinic effect was negligibly small. This type of spectrophotometer has proven ideally suited for detailed analysis of flash-induced absorbance changes at 515–520 nm (electrochromic shift) (Joliot and Delosme 1974; Joliot and Joliot 1989; Joliot et al. 2004), as well as of cyt b_6_f (Joliot and Joliot 1984, 1986, 1988) and of C-550 (Joliot and Joliot 1979). A first portable version for measurement with leaves was introduced by Kramer and Crofts 1990, which has been further developed over the past 20 years (see below).

A different kind of approach for measuring in vivo absorbance changes was taken by Klughammer et al. (1990), which was based on the Pulse-Amplitude-Modulation (PAM) method previously developed for measurements of chlorophyll fluorescence in natural daylight and assessment of various quenching parameters by the saturation pulse method (Schreiber 1986; Schreiber et al. 1986). This approach employs *continuous* trains of 1 μs ML pulses generated by light emitting diodes (LED), the frequency of which can be adjusted over a wide range (depending on the rate of the investigated changes), and a special pulse signal amplifier. The original spectrophotometer (Klughammer et al. 1990; Klughammer 1992) featured 16 independent monochromatic LED ML sources equipped with narrow band interference filters (530–600 nm), with the various wavelengths being sequentially pulsed at high-repetition rate. While the time resolution (1 ms) of this type of Kinetic LED Array Spectrophotometer (KLAS) cannot cope with that of the Joliot-type device (30 μs), the KLAS displays the practical advantage of absorbance being measured quasi-simultaneously at 16 wavelengths. In this way, changes can be measured continuously under close to natural conditions of illumination, during dark-light or light–dark induction and in the steady-state, very similar to chlorophyll fluorescence, rendering this device particularly suited for in vivo studies. The absorbance changes can be deconvoluted into the specific contributions of cyt f, cyt b-563, cyt b-559, and C550, as well as of changes caused by the electrochromic shift at 515–520 nm, “light scattering” around 535 nm and zeaxanthin at 505 nm (Klughammer et al. 1990; Klughammer 1992; Heimann 1998). So far practical applications of the KLAS have been quite limited, as only few prototypes were built by the authors (Ch.K. and U.Sch.) (for some examples of application see e.g., Klughammer and Schreiber 1993; Miyake et al. 1995; Heimann and Schreiber 1996; Klughammer et al. 1998; Aronsson et al. 2008; Miyake 2010; Takagi et al. 2012). A conceptually similar spectrophotometer allowing near-simultaneous measurements of absorbance changes at up to four different wavelengths was introduced by Avenson et al. (2004a) and described in more detail by Hall et al. (2012). Based on quasi-simultaneously measured 505, 520, and 535 nm single beam signals these authors differentiated ECS changes from overlapping changes of “light scattering” and zeaxanthin during continuous illumination by off-line deconvolution (Cruz et al. 2001).

During the past 10 years the KLAS has been further developed for measurements in the near-infrared and to support deconvolution of P700 and plastocyanin absorbance changes. Furthermore, in the 505–570 nm wavelength range now eight dual-wavelengths difference signals are measured quasi-simultaneously instead of 16 single beam signals, with the advantage that non-specific optical disturbances and signal changes are more effectively suppressed in the difference mode (Klughammer and Schreiber, in preparation). For measurements of rapid ECS (P515) changes, only one of the eight dual-wavelengths channels can be used, with a corresponding increase of time resolution (now 30 μs). The commercially available Dual-PAM-100, with which the measurements of the present study were carried out, is equivalent to a one channel dual-wavelength KLAS combined with a PAM fluorometer. While the basic version of this device measures the 870–820 nm dual-wavelength difference signal (P700), we have developed an accessory emitter–detector module optimized for measuring the 550–520 nm dual-wavelength difference signal (ECS and P515) simultaneously with the single beam 535 nm signal (“light scattering”) instead of Chl fluorescence (Schreiber and Klughammer 2008). Here we will concentrate on the ECS (P515) signal and on the charge-flux information carried by this signal upon rapid modulation of the actinic light. Our study builds on extensive previous work by Joliot, Kramer and co-workers on dark-interval relaxation kinetics (DIRK) of P515 (ECS), which not only contain information on the pmf and its partitioning into its ΔpH and Δ*Ψ* components (Sacksteder and Kramer 2000; Cruz et al. 2001), but also on the light-driven charge flux (Joliot and Joliot 2002; Kramer et al. 2004a, b; Joliot and Joliot 2006; Takizawa et al. 2007; Livingston et al. 2010). We will report on a special “flux mode” of Dual-PAM-100 operation, involving 1:1 light:dark modulation of AL on top of pulse amplitude modulation of the two ML beams. It will be shown that the “P515 flux” signal provides a reliable *continuous* measure of light-driven charge fluxes in photosynthesis, correlating well with simultaneously measured CO_2_ uptake in intact leaves. Deviations between the two signals can be interpreted in terms of alternative types of electron flow, regulatory changes in the conductivity of the reversible ATP synthase or of the H^+^/*e* ^−^ ratio (see Kramer et al. 2004a, b for a reviews).

## Materials and methods

### Experimental setup for simultaneous measurements of P515 and CO_2_ uptake

**Fig. 1:**
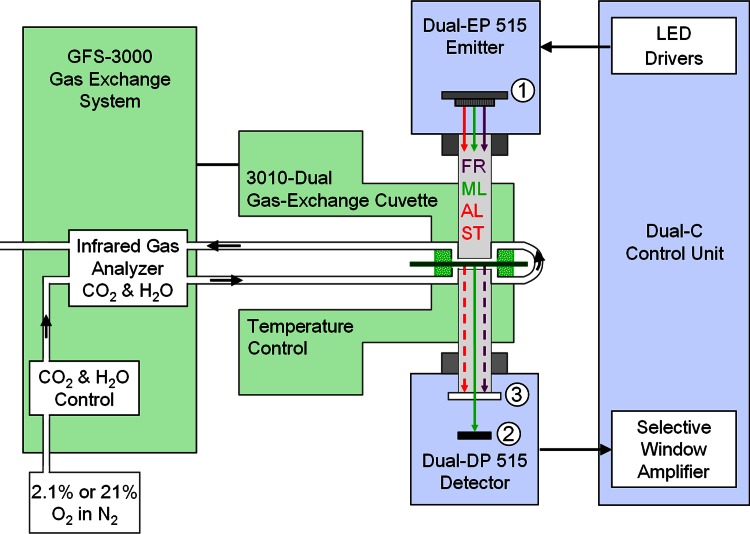
Block scheme of experimental setup for simultaneous measurements of dual-wavelength (550–520 nm) difference signal (P515) and CO_2_ uptake. For further explanations, see text

Experiments involving simultaneous measurements of P515 and CO_2_ uptake (Figs. 8, 9, 10) were carried out under controlled conditions of gas composition and temperature. A Dual-PAM-100 measuring system was combined with a GFS-3000 gas exchange measuring system. The Dual-PAM-100 and the dual-wavelength P515 module were developed by two of the authors (Ch.K. and U.Sch.). Both systems are commercially available (Heinz Walz GmbH, Germany). The experimental setup is depicted schematically in Fig. 1.

The leaf was enclosed in a gas-exchange cuvette (3010-DUAL, Walz), with an illuminated area of 1.3 cm^2^ and 1 mm chamber depth. Leaf temperature was kept close to 20 °C (between 19.5 and 21.5 °C). Within the cuvette the leaf was sandwiched between the end-pieces of two 10 × 10 mm perspex light guides connected to emitter (DUAL EP515) and detector (DUAL DP515) units of the Dual-PAM-100. CO_2_ and H_2_O concentration of the incoming gas was controlled via the GFS-3000 Gas Exchange System. A carrier gas with 2.1 % O_2_ in N_2_ was provided. The gas stream (400 μmol s^−1^) passed the leaf twice, at lower and upper sides before entering the Infrared Gas Analyzer for assessment of CO_2_-uptake and H_2_O-release. The emitter unit consisted of an array of 8 white LEDs equipped with interference filters. While the “550 nm” ML was derived from 3 white LEDs with 3 individual 550 nm interference filters (resulting wavelength 550.5 nm, 5.5 nm HBW), 4 white LEDs equipped with 4 individual 520 nm interference filters (resulting wavelength 518.5 nm, 8.5 nm HBW) provided “520 nm” ML. A single white LED with a 535 nm interference filter (5.5 nm HBW) gave 535 nm ML (not used for the measurements presented in this study). The 8 LEDs were arranged in a ring and focused via a central 6.5 mm hole in a chip-on-board (COB) LED array (featuring 635 nm Power-LEDs for actinic illumination) on a 10 × 10 mm Perspex rod, which served for mixing the various light qualities and guiding the randomized light to the leaf sample. In addition, a single 730 nm LED equipped with a 1 mm RG9 filter in the center of the LED array served for far-red illumination (FR). The COB array consisted of 24 Power-LED-Chips which for short times can be driven with high currents (up to 1.5 A). It provided not only continuous actinic illumination, but also saturating single turnover flashes (ST). The LED array (1) was powered by LED drivers in the DUAL-C control unit, containing dedicated hard- and firm-ware. The pulse-modulated green ML originating from the emitter unit was partially transmitted via the leaf into the outgoing 10 × 10 mm perspex rod and guided to the detector unit. Before reaching the 10 × 10 mm PIN-photodiode (2), it passed a blue-green filter (3) (1 mm BG39, Schott), which served for absorption of AL, ST, and FR lights. After pre-amplification, the pulse-modulated difference signal was processed with the help of a selective window amplifier within the DUAL-C control unit. Two settings of hardware damping of the signal were provided for fast and slow kinetics measurements, with 10 μs and 1 ms time constants, respectively. Saved data could be further processed by point averaging (software damping). The software supported repetitive measurements with on-line and off-line averaging. For further details of the P515 module, see Schreiber and Klughammer (2008).

### Details of the gas exchange measurements

Before measurement of each CO_2_- or light-curve the leaf was first kept in 380 μmol mol^−1^ CO_2_ and high light (1,120 μmol m^−2^ s^−1^) until the stomata-opening reached a steady state (conductance for H_2_O: 150–200 mmol m^−2^ s^−1^). When the leaf was acclimated to darkness before the measurement, the light was increased stepwise starting from 300 μmol m^−2^ s^−1^ to avoid photoinhibition. Humidity was additionally measured with a dew point mirror MTS-MK (Walz, Effeltrich, Germany), since the O_2_ concentration influences the infra red signal of H_2_O in the gas analyzer. The sum of assimilatory CO_2_ uptake (A) and CO_2_ released by day respiration (Resp) was used in this study.

### Measurements of P515 without simultaneous assessment of CO_2_ uptake

Experiments without simultaneous measurements of gas exchange were carried out at room temperature (20–22 °C) in ambient air. Leaves attached to well-watered potted plants were enclosed in the standard leaf-holder of the Dual-PAM-100 measuring system (see Fig. 1 in Schreiber and Klughammer 2008), with 1-mm distance between the perspex end pieces of the emitter and detector units. A constant stream of air (200 ml/min) was passed over the leaf.

### Plant material

Measurements were carried out with attached healthy leaves of well-watered potted plants of tobacco (*Nicotiana tabacum*) and dandelion (*Taraxacum officinale*). The plants were grown in natural daylight on the sill of a north window at light intensities between 50 and 150 μmol m^−2^ s^−1^. Dandelion plants (*Taraxacum officinale*) used for simultaneous measurements of gas exchange and P515 were grown in full day light (garden site) and potted 2–3 days before measurements in late autumn.

### Properties of the dual-beam 550–520 nm difference signal

The P515 signal was measured dual-beam as “550–520 nm” difference signal. As outlined above (under “Experimental setup for simultaneous measurements of P515 and CO_2_ uptake” section) the wavelengths of 550 and 520 nm correspond to the transmission peaks of the applied interference filters. In conjunction with the white LEDs, the actual wavelengths were 550.5 and 518.5 nm. Using white LEDs instead of green LEDs with predominant emission around 550 and 520 nm proved advantageous for minimizing temperature dependent drifts of the difference signal. The 550 nm reference wavelength was chosen in order to minimize the contribution of “light scattering” changes to the difference signal. The symmetrical Gauss-shape absorbance peak at 535 nm features a half-band width of about 26 nm, with absorbance being equally dropped to about 30 % both at 518.5 and 550.5 nm, so that the absorbance changes due to the 535 nm change should be about equal at 518.5 and 550.5 nm, i.e., canceling each other in the difference signal. This was confirmed by measurements with heat-treated leaves, which showed a strongly enhanced light-induced 535 nm change, whereas the simultaneously measured 550–520 nm difference signal was diminished (Schreiber and Klughammer 2008). Mild heat stress is known to stimulate “light scattering” and to suppress P515 (Bilger and Schreiber 1990). The chosen dual-wavelength difference approach has the advantage that P515 changes practically free of contamination by “scattering” changes can be measured directly on-line, whereas multi-wavelength single beam measurements (Avenson et al. 2004a; Hall et al. 2012) require off-line deconvolution.

The 550–520 nm dual-wavelength measurement does *not* eliminate a contribution of zeaxanthin changes to the P515 signal, as zeaxanthin absorption is distinctly higher at 520 nm compared to 550 nm (Yamamoto et al. 1972; Bilger et al. 1989). However, field indicating changes of P515 can be distinguished from changes due to zeaxanthin by their much faster responses. While following a saturating single-turnover flash the former shows pronounced changes in the sub-ms, ms, and s time ranges, the latter does not show any response to a brief flash and the changes induced by continuous illumination display response time constants in the order of minutes. Hence, the flash response can be taken as a specific measure of the field indicating electrochromic shift at 515–520 nm (see Fig. 5 below).

The Dual-PAM-100, with which the 550–520 nm absorbance changes were measured, employs a special modulation technique for dual-wavelength measurements, conceived for high flexibility of ML pulse frequency, with the purpose to prevent significant sample pre-illumination without sacrificing time resolution and signal/noise ratio. The ML pulses are applied in the form of 30 μs “pulse blocks” (with each block containing 12 pulses) separated by variable dark times. “Low block frequencies” from 1 to 1,000 Hz are provided for monitoring the signal with negligibly small actinic effect. Simultaneously with onset of actinic illumination “High block frequency” can be applied (up to 20 kHz), so that light-induced changes are measured with high-time resolution and signal/noise ratio. At a “block frequency” of 20 kHz there is no dark time between the “pulse blocks”, which means continuous pulse modulation at 200 kHz for monitoring the difference signal. Time integrated ML intensity (at *maximal* intensity setting) amounted to 0.06 μmol m^−2^ s^−1^ at 200 Hz “block frequency” (applied for measuring baseline signal before actinic illumination) and 6.3 μmol m^−2^ s^−1^ at *maximal* “block frequency” of 20 kHz. For measurement of flash-induced changes the ML was triggered on at maximal frequency 100 μs before triggering of the flash. In this way, a pre-illumination effect could be completely avoided.

With each new sample, the LED currents of the two ML beams were adjusted with the help of an automated routine such that the difference signal was close to zero. Single beam signals were in the order of 10–30 V. After balancing the two signals, the difference signal could be strongly amplified without risk of amplifier saturation. The amplitude of the single signals (corresponding to *I*), which may be more than 1,000× larger than the recorded signal changes (corresponding to Δ*I*), were determined with the help of a special calibration routine, involving a defined transient decrease of the 520 nm signal with respect to the 550 nm signal (via corresponding decrease in LED current). The original difference signals were measured in Volt units, which were transformed into Δ*I*/*I* units by the calibration.

The long-term stability of the dual-beam difference signal was tested with the help of an “artificial leaf” consisting of a plastic filter sheet with a transmittance spectrum in the green region similar to that of a green leaf (Roscolux #01, Light Amber Bastard). Signal stability was best at relatively low frequency of the pulse-modulated ML (less than 10^−4^ Δ*I*/*I* units drift over a 5-min time period at frequencies up to 1 kHz). On the other hand, for measurements of flash-induced rapid changes maximal pulse modulation frequency of 200 kHz was used, where the signal/noise is optimal and the drift (approximately 2 × 10^−3^ Δ*I*/*I* units drift over a 5-min time period) does not affect measurements in the s time range. Maximal pulse modulation frequency of 200 kHz was also applied for the flux measurements described under “Results and discussion” section, where not only the ML, but also the AL is modulated.

## Results and discussion

### Partitioning of total pmf between ΔpH and Δ*Ψ* in tobacco leaves

Analysis of DIRK method has been advanced by Kramer and co-workers for non-intrusive measurement of the rate of electron flow via P700 (Sacksteder and Kramer 2000), for assessment of the ΔpH and Δ*Ψ* components of overall pmf (Cruz et al. 2001; Avenson et al. 2004a) and for determination of the rate of proton efflux via the ATP-synthase (Sacksteder et al. 2000; Kanazawa and Kramer 2002; Kramer et al. 2003; Cruz et al. 2005). Most of this previous work has been based on single beam absorbance measurements of the ECS around 515–520 nm. In order to minimize problems arising from overlapping “light scattering” changes (peaking at 535 nm) a diffused-optics spectrophotometer (Kramer and Sacksteder 1998) or non-focusing optics spectrophotometer (Sacksteder et al. 2001) were used. In our P515 measuring system “light scattering” changes are largely eliminated by the dual-wavelength (550–520 nm) approach (Schreiber and Klughammer 2008, see also corresponding section under “Materials and methods” section). While the dual-wavelength technique does *not* eliminate changes due to zeaxanthin (peaking around 505 nm), such changes are unlikely to contribute to dark-induced relaxation kinetics, as they are very slow and, hence, can be readily distinguished from the much more rapid ECS changes analyzed by the DIRK method. In our measuring system, a long-term dark-adapted tobacco leaf attached to a well-watered potted plant displays a stable baseline for hours. Upon exposure to continuous illumination, complex induction kinetics are observed that reflect genuine changes of the membrane potential as well as a slow continuous rise due to zeaxanthin formation, the extent of which depends on light intensity (see e.g., Fig. 11 in Schreiber and Klughammer 2008). The relative extent of overlapping zeaxanthin changes can be minimized by pre-illuminating the leaf for about 40 min at relatively high irradiance (e.g., 600 μmol m^−2^ s^−1^) to fill up the zeaxanthin pool.

**Fig. 2:**
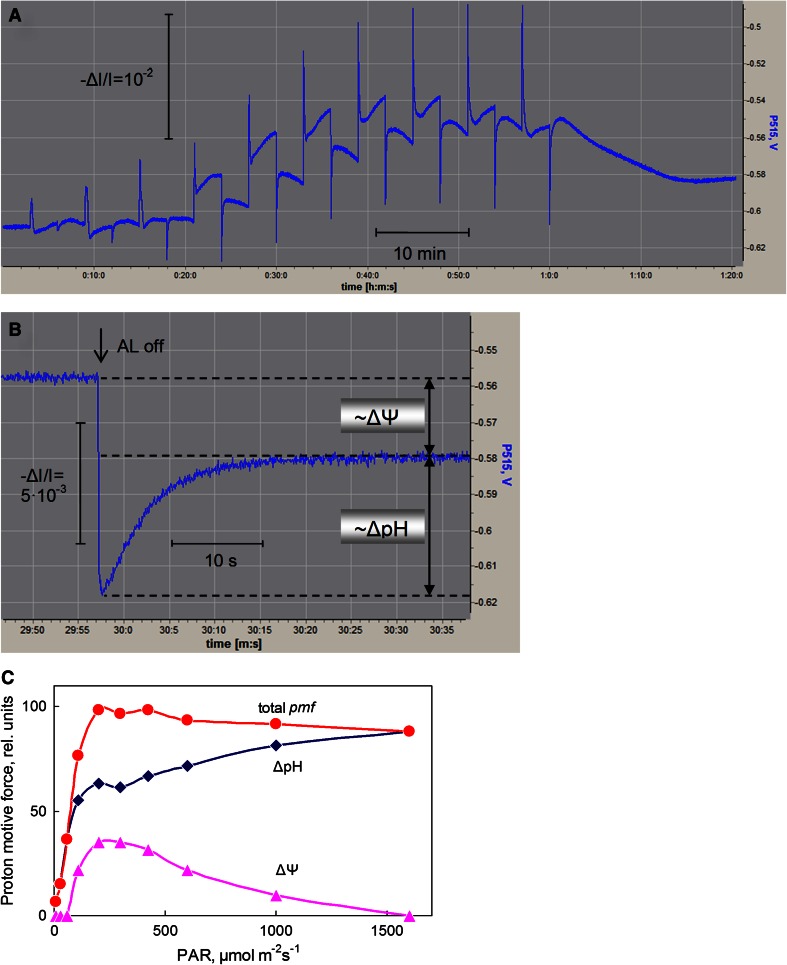
Repetitive application of the DIRK method during an increasing light response curve of a tobacco leaf. **a** Change of the dual-wavelength (550–520 nm) difference signal (P515) of the pre-illuminated leaf during the course of ten 3 min illumination periods at increasing light intensities, separated by 3 min dark periods, with the dark-interval relaxation kinetics following each illumination period serving for determination of the ΔpH and Δ*Ψ* components of the overall pmf. Actinic intensity was increased in ten steps from 10 to 1,600 μmol quanta m^−2 ^s^−1^ of 635 nm light. Leaf pre-illuminated for 1 h at 600 μmol m^−2 ^s^−1^, with 10 min dark time before start of recording. Screenshot of the original recording (Dual-PAM user software). **b** Deconvolution of the ΔpH and Δ*Ψ* components of the overall pmf by the DIRK method. Zoomed detail of the data set presented in **a**, showing dark-interval relaxation kinetics after turning off 200 μmol m^−2 ^s^−1^ (light step 5 in **a**). **c** Partitioning of overall proton motive force (pmf) into ΔpH and Δ*Ψ* components as a function of light intensity during the course of the experiment depicted in **a**. ΔpH and Δ*Ψ* were determined as explained in **b**

An experiment analogous to that depicted in Fig. 11 of Schreiber and Klughammer (2008) is presented in Fig. 2a, with the difference that the leaf had been pre-illuminated before start of the recording, so that zeaxanthin changes were minimized. The experiment involved ten consecutive DIRK measurements of the ΔpH and Δ*Ψ* components of pmf after adjustment of the photosynthetic apparatus to stepwise increasing light intensities. With each light-on of the various intensities, complex induction transients were observed consisting of rapid positive spikes followed by slower rise phases. Conversely, with each light-off there were rapid negative spikes that were followed by slow rise phases to transient peaks and consequent slow declines. For DIRK analysis the amplitude of the rapid light-off response and the level of the slow light-off peak are decisive. The principle of this method is outlined in Fig. 2b, which shows a zoomed detail of the data in Fig. 2a, namely DIRK analysis of the quasi-stationary state reached after 3 min exposure to 200 μmol m^−2^ s^−1^ (light step 5). The rapid negative change reflects the overall pmf in the given state and the slow peak level defines the partition line between ΔpH and Δ*Ψ* components (Cruz et al. 2001). Under the given conditions, at 200 μmol m^−2^ s^−1^ the Δ*Ψ* component contributes about 1/3 to the overall pmf. The light-intensity dependence of partitioning between ΔpH and Δ*Ψ* is depicted in Fig. 2c. At low intensities (up to about 60 μmol m^−2^ s^−1^) the Δ*Ψ* component was negligibly small, while the ΔpH component had already reached about 1/3 of its maximal value. A peak of Δ*Ψ* was observed at 200 μmol m^−2^ s^−1^, which was paralleled by a transient peak in ΔpH. Interestingly, with further increasing intensities there was a further *increase* of ΔpH correlating with a *decrease* of Δ*Ψ*. Hence, at higher light intensities there seems to be transformation of Δ*Ψ* into ΔpH, without much change in the total pmf (Fig. 2). The overall pmf was found to peak between 200 and 400 μmol m^−2^ s^−1^, decreasing by about 10 % when light intensity was further increased to 1,600 μmol m^−2^ s^−1^.

As has been discussed extensively by Kramer and co-workers (for reviews see Kramer et al. 2004a, b; Cruz et al. 2004; Avenson et al. 2005b), the pmf and its ΔpH and Δ*Ψ* components play a dual role in photosynthesis, namely at the level of energy transduction (synthesis of ATP from ADP and P_i_ at the thylakoid CF_0_–CF_1_ ATP synthase) and at the level of regulation. In particular, the ΔpH has been known to regulate the efficiency of light capture in PS II via dissipation of excess energy, which otherwise would lead to photodamage (Demmig-Adams 1992; Niyogi 1999). The observed increase of the ΔpH component above 300 μmol m^−2^ s^−1^ on the cost of the Δ*Ψ* component (Fig. 2c) may serve as an example for the adaptive flexibility of the photosynthetic apparatus. While Δ*Ψ* contributes substantially to overall pmf at moderate PAR, where the efficiency of light capture is decisive, maximal ΔpH is approached at high light intensities only, where down-regulation of PS II becomes essential.

Very recently Johnson and Ruban (2013) questioned the existence of a substantial Δ*Ψ* components in plant leaves during steady-state illumination, as suggested by Kramer and co-workers, on the grounds of experiments with nigericin-infiltrated leaves of wild-type *Arabidopsis* and with leaves of *Arabidopsis* mutants deficient in energy-dependent fluorescence quenching (qE). These authors argue that the apparent ECS in normal leaves during steady-state illumination is not due to a genuine 515 nm change, i.e., is not caused by Δ*Ψ*, but in fact reflects an overlapping qE-related absorption change, the position of which varies depending on the xanthophyll content of the leaves between 525 and 540 nm (Johnson et al. 2009). It may be pointed out that all measurements of Johnson and Ruban (2013) were carried out using 700 μmol m^−2^ s^−1^ red light, i.e., at a high intensity of absorbed light, where also our data show a rather small Δ*Ψ* component (Fig. 2c). The decrease of Δ*Ψ* at high intensity could be due to enhanced influx of anions (Cl^−^) and efflux of cations (Mg^++^) accompanying the light-driven influx of protons into the thylakoid lumen (Hind et al. 1974). As suggested by Johnson and Ruban (2013) also voltage-gated anion (Schönknecht et al. 1988) and cation (Pottosin and Schönknecht 1996) channels could be involved.

### Fast DIRK recording and new technique of continuously measured charge flux

**Fig. 3:**
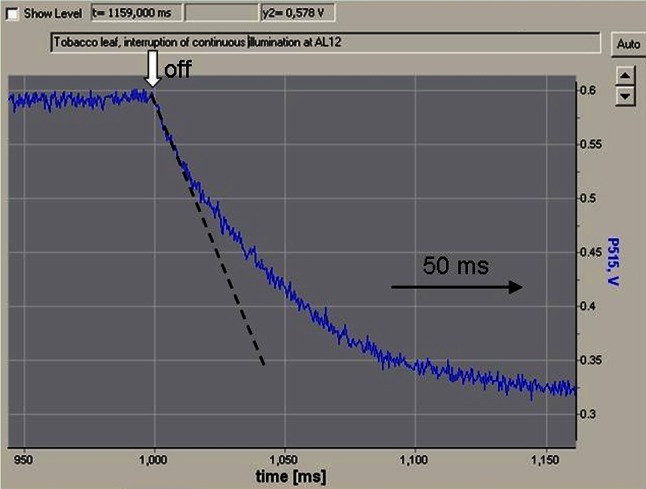
Recording of the fast decay phase of the DIRK_ECS_ response with indication of the initial slope reflecting the rate of charge flux briefly before light-off

For the DIRK analysis demonstrated in Fig. 2b the P515 signal was recorded with a time resolution of 10 ms/point, which is more than sufficient to determine the amplitude of the rapid negative transient peaking around 350 ms after light-off. A much higher time resolution is required to resolve the *initial kinetics* of the rapid negative transient. Figure 3 shows a screenshot of a recording with 0.1 ms/point resolution (Fig. 3).

The *initial* *slope* of the dark-interval ECS-decay carries twofold information on the rate of photosynthetic charge fluxes, in terms of both electron and proton transport (Cruz et al. 2001; Sacksteder et al. 2001; Joliot and Joliot 2002; Joliot et al. 2004). Light-driven vectorial electron transport is coupled with proton transport from the stroma to the lumen, which is balanced by proton efflux via the ATP synthase, so that ECS in a quasi-stationary state is constant (zero rate of ECS change, *R*_light_ = 0). Upon light-off, the light-driven reactions stop, whereas proton efflux continues in the dark. Furthermore, it has to be considered that the light-driven electrogenic reactions not only involve charge separation at PS II and PS I, but also vectorial proton translocation from the stroma to the lumen in the Q-cycle at the cyt b_6_f complex (Velthuys 1978). If it is assumed that the rate of the Q-cycle is not appreciably changed during the first ms after light-off (Joliot and Joliot 2002), it follows for the ECS changes in a quasi-stationary light state briefly before and after light-off, *R*_light_ and *R*_dark_, respectively (Joliot et al. 2004):

1. *R*_light_ is proportional to *R*_ph_ + *R*_bf_ − *R*_efflux_, with *R*_ph_ being the overall rate of photochemical charge separation in PS I and PS II, *R*_bf_ the rate of proton translocation coupled with cyt bf turnover and *R*_efflux_ the rate of proton efflux via the ATP synthase.
2. *R*_dark_ is proportional to *R*_bf_ − *R*_efflux_, as *R*_ph_ = 0.
3. *R*_light_ − R_dark_ is proportional to *R*_ph_ + *R*_bf_ − *R*_efflux_ − (*R*_bf_ − *R*_efflux_) = *R*_ph_.

If in a quasi stationary light state positive and negative electrogenic reactions are balanced, as in the experiment of Fig. 3, *R*_light_ = 0 and *R*_dark_ is directly proportional to *R*_ph_. Furthermore, *R*_dark_ is also a measure of the rate of proton efflux via the ATP ase, i.e., proportional to the rate of ATP synthesis. However, as apparent from point (2) above, the proportionality only holds as long as it is assumed that the Q-cycle is obligatory (Sacksteder et al. 2000). Furthermore, any attempt to estimate *absolute* rates of proton efflux and consequently of ATP formation, has to take into account that *R*_dark_ is lowered with respect to *R*_efflux_ by *R*_bf_, which would represent 1/3 of overall proton influx, if the Q-cycle is obligatory.

Based on fast DIRK recordings as shown in Fig. 3, it is possible to obtain point-by-point information on the rate of coupled electron transport, e.g., as a function of light intensity (Sacksteder et al. 2001) or during dark-light induction (Joliot and Joliot 2002; Joliot et al. 2004). While this approach provides straight-forward information, it is time consuming and cumbersome, as for each recording the initial slope after light-off has to be evaluated. Furthermore, for comparison of several data points, e.g., during dark-light induction, it is essential that all measurements are carried out under close to identical conditions, particularly in terms of the state of pre-illumination, which is not always easy.

We have developed a somewhat different technique which provides a *continuous* measure of the same charge flux (*R*_dark_) that can be measured point by point via the initial slope of the DIRK response. An analogous technique previously has been described for continuous monitoring of electron flux via PS I (P700 flux method, Klughammer 1992). This technique is based on a 1:1 light:dark modulation of the actinic light. The light/dark periods can be varied among 1, 2, 5, 10, 20, and 50 ms. Light/dark periods of 2–5 ms proved optimal in terms of signal amplitude and signal/noise ratio. During the light periods, the P515 indicated membrane potential (pmf) increases (via charge separation in the two photosystems and vectorial proton flux associated with the Q-cycle) and during the dark periods the P515 indicated pmf decreases again (primarily due to proton efflux via the ATP synthase).

**Fig. 4:**
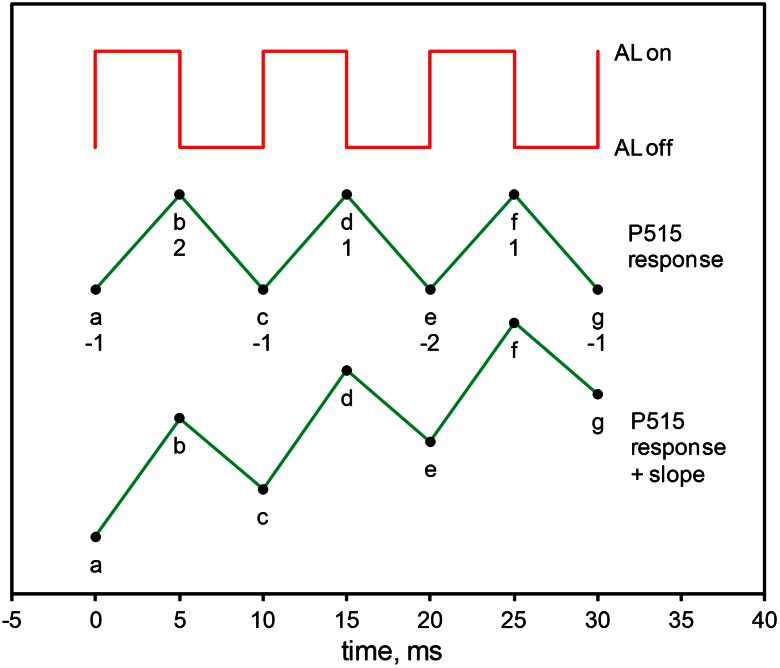
P515 signal changes (*triangular responses*) in response to 1:1 light:dark modulated actinic light depicted schematically for a stable signal (*top*) and a sloping signal (*bottom*). From the amplitudes of the triangular responses a continuous flux signal is derived, as explained in the text. *Note* using the approach described in the text, with and without slope the same flux signal results

**Fig. 5:**
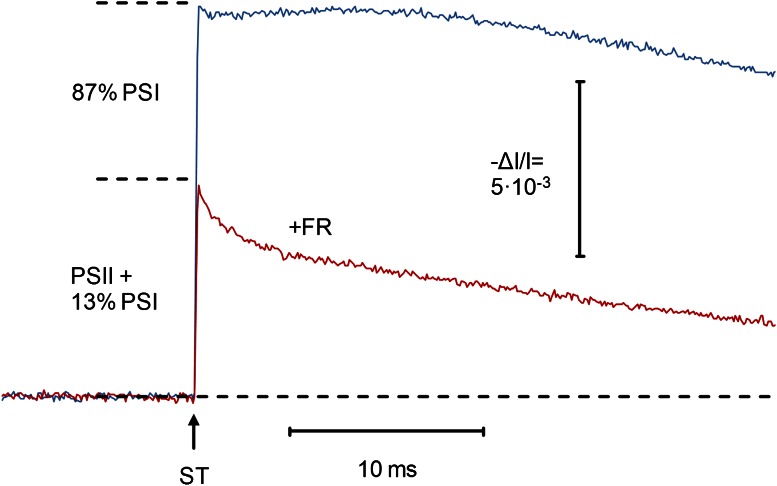
Flash-induced P515 changes of a dandelion leaf in the absence (*blue curve*) and the presence (*pink*) of FR background light (intensity step 5). The amplitudes of the fast phases were determined by extrapolation to time zero. Flash intensity was saturating at the chosen width of 40 μs as verified by separate measurements (not shown). 50 averages each

In Fig. 4 the principle of generation of the P515 indicated flow signal (*R*_dark_) is depicted schematically for 5 ms light/dark periods. Modulation of the red actinic light at 200 Hz is synchronized with sampling of the P515 dual-wavelength difference signal (black points). In the flux mode, the dual-wavelength ML is modulated at maximal frequency of 200 kHz (see “Materials and methods” section), resulting in a continuous signal after pulse amplification. This signal can be “sampled” with 1, 2, 5, 10, 20 ms/point, etc., depending on the setting of acquisition rate in the user software of the Dual-PAM-100. In the example of Fig. 5, a 5 ms sampling rate was used. Within the depicted 5-ms time intervals positive and negative charge displacements corresponding to the P515 changes from a to b to c, etc. are measured. While in principle the charge flow signal could be simply derived from the signal values (b − a), (d − c), (f − e), etc. and division by Δ*t*, a different approach was applied in order to avoid artifacts under non-steady state conditions, i.e., when changes in the P515 signal during individual dark/light periods may be significant. The effect of sloping P515 signals was eliminated by subtracting the negative slopes from the preceding positive slopes, respectively. In practice, the P515 signal values were multiplied by the factors indicated under a, b, c, etc. in Fig. 4, three values each were added and divided by 2 × Δ*t*:

$$ {\text{flow rate}}\,(t1) = \frac{b - a + b - c}{2 \cdot \Updelta t} = \frac{ - a + 2 \cdot b - c}{2 \cdot \Updelta t} = \frac{{b - \frac{a + c}{2}}}{\Updelta t} $$

$$ {\text{flow rate}}\,(t2) = \frac{d - e + f - e}{2 \cdot \Updelta t} = \frac{d - 2 \cdot e + f}{2 \cdot \Updelta t} = \frac{{\frac{d + f}{2} - e}}{\Updelta t} $$

etc.

The advantage of this approach is apparent from the example of a measurement with positively sloping P515 signal in Fig. 4. In the given case, using the simple approach the flow rate would be overestimated by 22 %, whereas the flow rate determined with the approach outlined above is not affected by the slope. Another advantage of this approach is that any non-modulated change of the P515 signal, as e.g., occurring when the actinic light is switched off permanently, does not lead to artefacts and negative flow signals.

### Quantification of the charge flux signal

The original charge flux data consist of changes of the dual-wavelength (550–520 nm) Δ*I*/*I* with time, i.e., rates of *relative* changes in transmission. In order to obtain *absolute* estimates of charge flux rates that can be compared with e.g., PS II turnover, Δ*I*/*I* has to be calibrated. In principle, the Δ*I*/*I* corresponding to a single charge separation in PS II can be determined with the help of single turnover saturating flash (ST) measurements. Such measurements require high sensitivity and time resolution. They are complicated by the fact that a 40–50 μs flash, which in our P515 measuring system is required for a saturated single turnover of PS II in leaves, may cause more than one turnover in PS I. Furthermore, the PS II/PS I ratio is not known. These complications were overcome by pre-oxidizing P700 using FR background light so that most of the ST-induced Δ*I*/*I* due to PS I turnover was suppressed. Parallel P700 measurements carried out with the same leaf under identical conditions revealed a 13 % fraction of P700 that was not oxidized by the FR (data not shown). Based on this information, an accurate determination of the Δ*I*/*I* corresponding to a single turnover of PS II was possible, as illustrated in Fig. 5.

The fast P515 change caused by PSII only, P515(PSII), was calculated as follows:

$$ {\text{P}}515\left( {\text{PSII}} \right) = \frac{{{\text{P}}515\left( {\text{FR}} \right) - n \cdot P515}}{1 - n} = \frac{{(6.21 - 0.13 \cdot 11.27) \times 10^{ - 3} }}{1 - 0.13} = 5.45 \times 10^{ - 3} $$

where *n* = 0.13 is the non-oxidized part of P700, and P515 and P515(FR) are the fast P515 changes in absence and presence of FR light, respectively.

### Performance of the charge flux signal in slow kinetics measurement

**Fig. 6:**
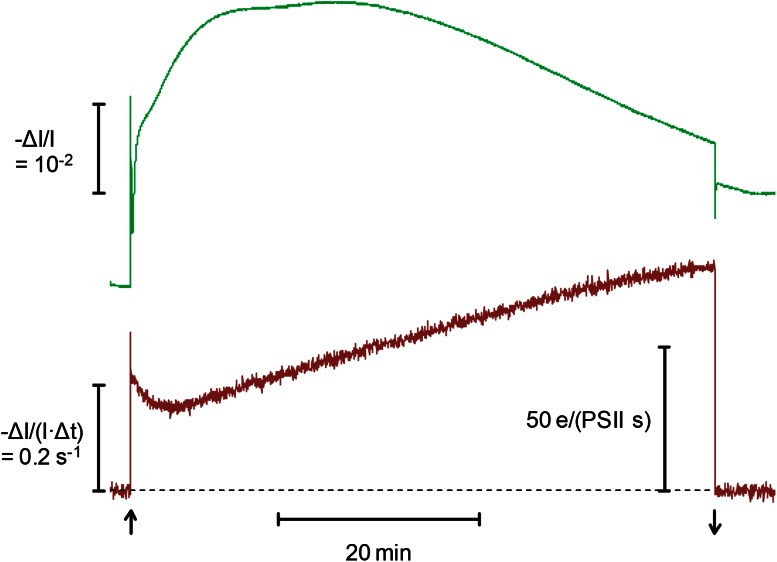
Simultaneous recordings of original P515 signal (ECS) (top curve) and P515 indicated charge flux signal (*bottom curve*) during dark-light induction of a dandelion leaf. Time integrated light intensity, 635 μmol m^−2 ^s^−1^. Alternating 5 ms light and 5 ms dark periods, as explained in Fig. 4

Figure 6 (bottom curve) shows an example of a dark-light induction curve of P515 signaled charge flux (*R*_dark)_. The charge flux rate originally measured in units of Δ*I*/(*I* × Δ*t*) s^−1^ (i.e., from the P515 response during 5 ms light–dark periods) is also indicated in absolute units of electrons per s and PS II, using the calibration factor of 5.45 × 10^−3^ derived in Fig. 5 (i.e., the Δ*I*/*I* corresponding to one charge-separation at PS II). The simultaneously measured P515 signal, from which the charge flux signal was derived (see Fig. 4) is also depicted (top curve). It may be noted that the seemingly continuous P515 signal was hardly affected by the 5 ms dark-periods, during which *R*_dark_ was assessed. Hence, this signal may be considered close to identical to a signal measured with continuous actinic light at 50 % intensity (Fig. 6).

When the AL is switched off at the end of the 60 min illumination period, the DIRK information of pmf partitioning into Δ*Ψ* and ΔpH (see Fig. 2b for details) is also obtained in the flux mode of operation. As explained above (see text accompanying Fig. 2a), the slow changes of the P515 signal during dark-light induction not only reflect changes in the membrane potential, but of zeaxanthin as well. The apparent increase of the baseline is due to accumulation of zeaxanthin. On the other hand, the *flux signal* does *not* contain any contribution of zeaxanthin, as zeaxanthin does not respond to the 5 ms modulation of the AL. The same would also be true for any “contamination” of the P515 signal by a qE-related absorbance change, which may have to be considered according to recent findings of Johnson and Ruban (2013) (see discussion of Fig. 2 above).

When the charge flux signal is measured over longer periods of time using 5 ms light/dark intervals, as in the example of Fig. 6, extensive point averaging can be used (200–500 points), which results in satisfactory signal/noise in *single recordings*. This aspect is important for the simultaneous measurements of charge flux and CO_2_ uptake reported below, where averaging of several consecutive measurements would not be practicable.

### Comparison of new continuous flux approach with point-by-point DIRK approach

**Fig. 7:**
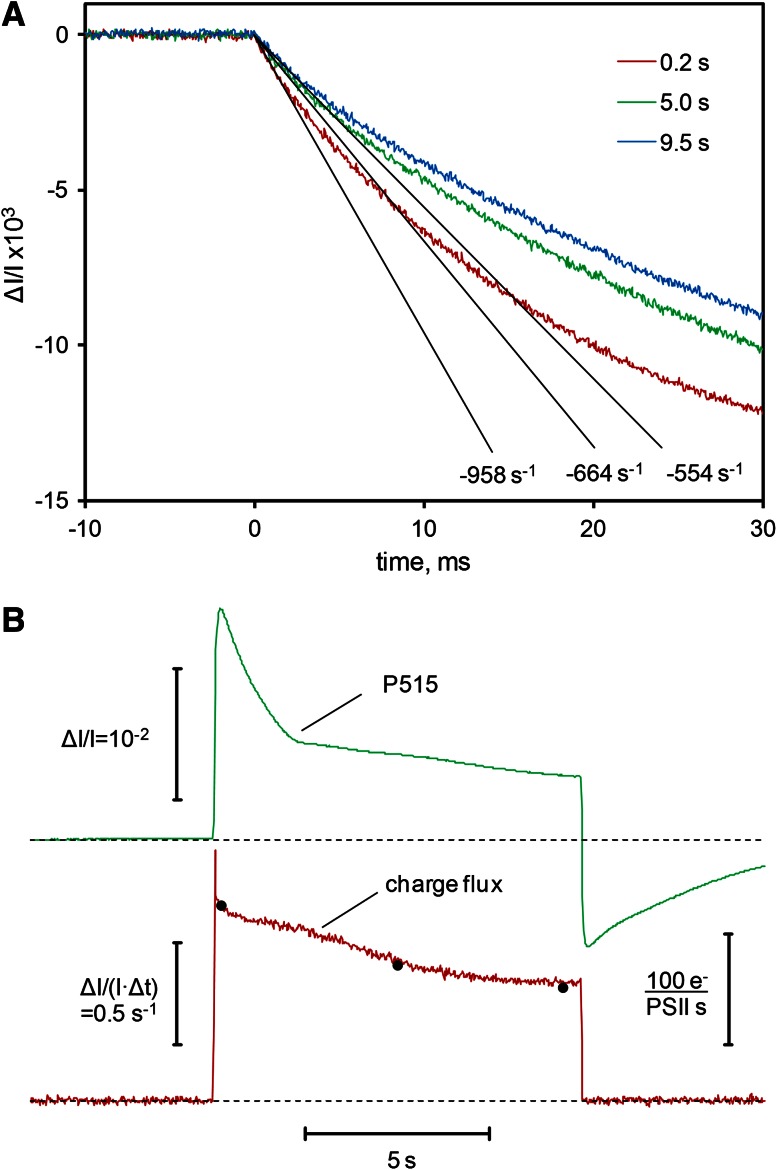
Comparison of continuous charge flux method with point-by-point DIRK_ECS_ method. **a** Determination of initial slopes of the ECS (P515) relaxation during 40 ms dark intervals for three points in the time course of repetitively measured dark-light induction curves (30 s repetition cycle) of a dandelion leaf. Average of 50 recordings. AL intensity, 1,920 μmol m^−2 ^s^−1^. **b** Dark-light induction curve of continuous charge flux signal (*bottom*) measured with the same leaf under close to identical conditions as in **a**. *Black points* rate of charge flux determined from initial slopes in **a** for comparison. P515 signal measured in the flux mode (*top*). Averages of 50 recordings. AL was 1:1 light:dark modulated with 2 ms on/off periods. Damping 10 μs. Average intensity, 1,920 μmol m^−2 ^s^−1^. For further explanations, see text

The potential of the point-by-point DIRK_ECS_ approach for obtaining in vivo information on the dynamic flexibility of photosynthetic charge fluxes has been demonstrated in numerous previous studies (Kramer and Sacksteder 1998; Cruz et al. 2001; Sacksteder et al. 2001; Joliot and Joliot 2002; Joliot et al. 2004; Avenson et al. 2004a). Therefore, for the acceptance of the new *continuous flux* approach it is important to show that the obtained information is equivalent to that provided by the proven DIRK_ECS_ method. Comparative measurements with both methods were carried out using the same leaf under close to identical conditions. For this purpose, the leaf was repetitively illuminated every 30 s for 10 s at 1,920 μmol m^−2^ s^−1^. When after 50 illumination cycles the kinetic response was constant, three DIRK_ECS_ data sets were recorded at times 0.2, 5.0, and 9.5 s after onset of actinic illumination, by measuring the fast decay kinetics during a 40 ms dark-period. Each data set consisted of 50 averages, all measured under the same repetitive regime of illumination. Figure 7a shows the resulting three decay curves with indication of the initial slopes, which were determined by linear regression using the data points of the first 2 ms after light-off only.

After having recorded the three DIRK_ECS_ data sets, the system was switched to flux mode and the actinic intensity was doubled, so that the average light intensity during 1:1 modulation again was 1,920 μmol m^−2^ s^−1^. Then the same repetitive regime of illumination was established and 50 illumination cycles were averaged in the flux mode with 2 ms on/off periods. Figure 7b shows the resulting charge flux induction curve (bottom) and also the simultaneously measured induction curve of the original P515 signal (top). The three black dots on top of the charge flux curve correspond to the initial slope data shown in Fig. 7a. Charge flux originally measured in units of Δ*I*/(*I* × Δ*t*) s^−1^ (i.e., the *P515* response during 2 ms light–dark periods) is also quantified in units of electrons per s and PS II, after transforming Δ*I*/(*I* × Δ*t*) into “PS II-related charge flux” using the calibration factor derived in Fig. 5 (i.e., Δ*I*/*I* = 5.45 × 10^−3^ for 1 *e* ^−^ per PS II). For example, the initial slope of Δ*I*/(*I* × Δ*t*) × 10^−3^ = 554 s^−1^ measured 9.5 s after light-on is equivalent to 102 *e* ^−^ per PS II and s. It should be noted that this “PS II-related charge flux” does *not* correspond to the actual PS II charge separation rate occurring in the given example at 9.5 s after light-on, but rather to the *overall rate* of photochemical charge separation in PS I and PS II (*R*_ph_, see definition above). If it were assumed that the rates of PS I and PS II are equal in a quasi-stationary state, the actual PS II charge separation rate would be 50 % of the “PS II-related charge flux”. However, electron flux rate via PS II would be less, if cyclic PS I would contribute to charge flux.

In the context of this technical report it is essential that almost identical charge flux rates are obtained with the point-by-point DIRK_ECS_ and the continuous P515 flux methods, with the latter having the obvious advantage of being less time consuming and more simple in practical applications. As the flux signal is quasi-continuous, its measurement does not disturb other continuously measured signals, like oxygen evolution or CO_2_ uptake. In the following sections simultaneous measurements of CO_2_ uptake and P515 indicated charge flux are presented.

### Comparison of CO_2_ uptake and charge flux: light response

**Fig. 8:**
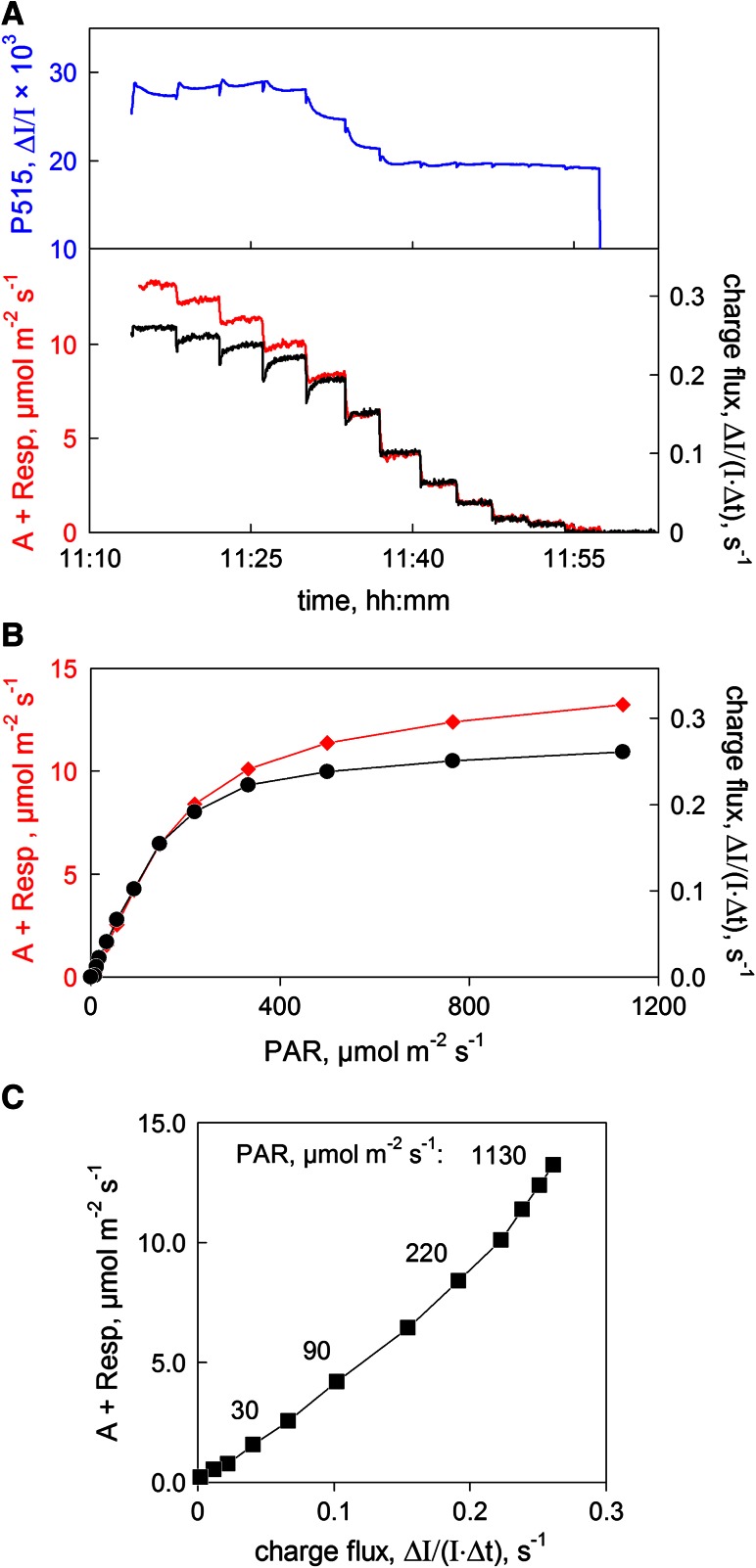
Simultaneously measured CO_2_ uptake (A + Resp) and P515 indicated charge flux in a dandelion leaf during the course of stepwise decrease of light intensity. Before start of measurement the leaf had been extensively pre-illuminated: 30 min at slowly increasing PAR up to 1,120 μmol m^−2 ^s^−1^ at 380 μmol CO_2_, followed by 50 min at 1,120 μmol m^−2 ^s^−1^, for stomatal opening and accumulation of zeaxanthin. 2.1 % O_2_ and 380 μmol mol^−1^ CO_2_ in nitrogen. 5 ms light/dark intervals. **a** Original recording of light-induced changes with the original P515 signal displayed at the top. Scaling of the charge flux trace adjusted to match the CO_2_ uptake trace in the low-intensity range. **b** Comparison of light response curves of P515 indicated charge flux and CO_2_ uptake. Based on original data in **a**. **c** Relationship between the rates of P515 indicated charge flux and CO_2_ uptake as a function of light intensity. Derived from the original data in **a**

Simultaneously measured changes of P515, P515 indicated charge flux and CO_2_ uptake induced by stepwise lowering of light intensity, are shown in Fig. 8a. P515 indicated charge flux is presented in units of Δ*I*/(*I* × Δ*t*) s^−1^, i.e., without information on PS II density, PS II/PS I and a possible contribution of cyclic PS I, no attempt was made to compare the rates of charge flux and CO_2_ uptake in absolute terms. The charge flux and CO_2_ uptake signals were scaled such that the responses in the low-intensity range were close to identical. At the same time the observed flux responses in the high-intensity range were relatively smaller, thus suggesting an earlier light saturation of charge flux compared with CO_2_ uptake, as evident in the light intensity plots (Fig. 8b). When plotted against each other (Fig. 8c), a curvi-linear relationship was apparent, with the deviation from linearity being small, at least up to about 200 μmol m^−2^ s^−1^.

As the CO_2_ uptake signal is a measure of the rate of linear electron transport (LEF) and the charge flux signal proportional to proton efflux via the ATP-synthase (as long as Q-cycle is obligatory), the slope of the *x*–*y* plot in Fig. 8c may be considered as a relative inverse measure of the H^+^/*e* ^−^ ratio of photosynthetic electron transport. Possibly, while being almost constant at light intensities up to approximately 200 μmol m^−2^ s^−1^, the H^+^/*e* ^−^ declines significantly at higher intensities. The simultaneously measured changes of the P515 signal, which under the given conditions (long-term pre-illuminated sample) should not show any significant zeaxanthin changes, suggest that in the same range of intensities where H^+^/*e* ^−^ declines, there is a large increase of the overall pmf. It may be speculated that a facultative pathway of coupled alternative (i.e., not CO_2_ reducing) electron transport either is controlled by the pmf or simply saturating at high PAR (e.g., “over-reduction” of a cyclic PS I electron transport chain). Alternatively, if the Q-cycle was facultative (Berry and Rumberg 1999), it could be suppressed when a certain pmf has been built up. These explanations, however, should be considered tentative, as they probably are not exclusive for the presented data.

While it is not possible to directly calculate an electron transport rate from the ECS-indicated proton-motive charge flux without detailed information on PS II/m^2^ and the PS I/PS II ratio, based on the observed curvi-linear relationship between charge flux and CO_2_ uptake signals, and calibration of the former by the latter, electron transport rates can be readily estimated from charge flux measurements.

### Comparison of CO_2_ uptake and charge flux: CO_2_ response curves

**Fig. 9:**
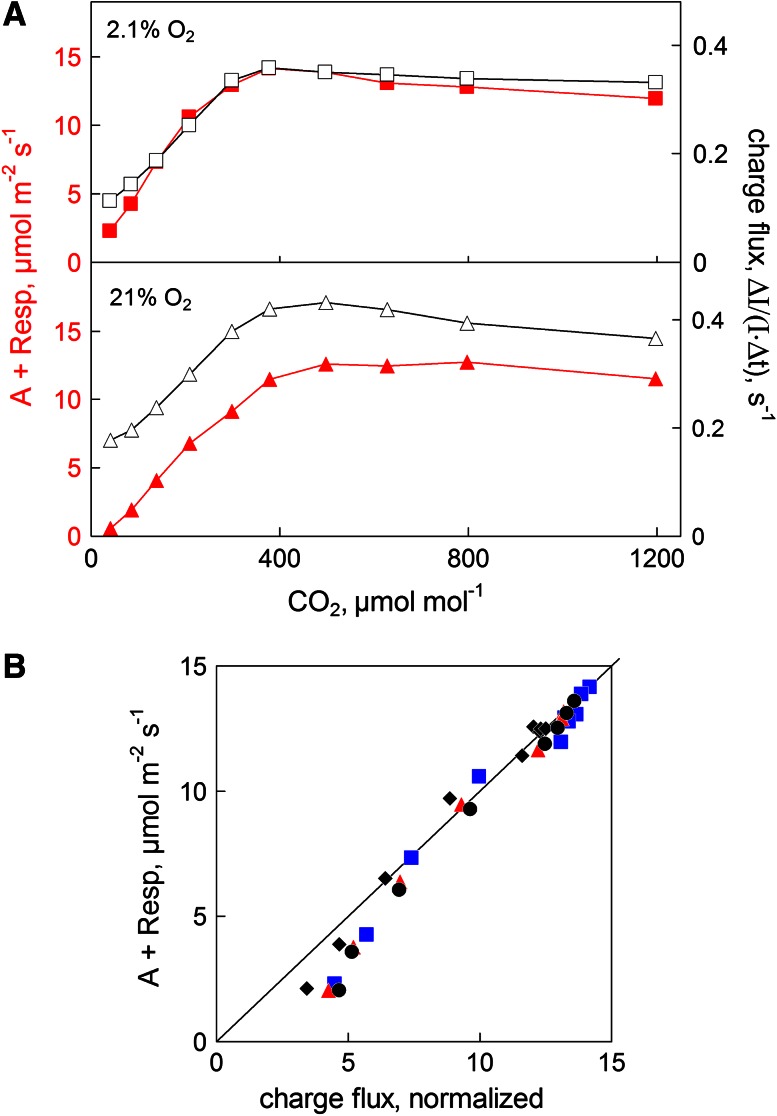
Comparison of CO_2_ uptake and P515 indicated charge flux as a function of CO_2_ concentration. Light intensity, 1,120 μmol m^−2 ^s^−1^. Attached dandelion leaf. 5 ms light/dark intervals. **a** Plots of the two signals versus CO_2_ concentration for 2.1 and 21 % O_2_. **b** Relationship between the rates of CO_2_ uptake and charge flux as a function of CO_2_ concentration in three different dandelion leaves at 2.1 % O_2_. The *symbols* represent *black diamonds*, leaf 1, 5 ms light/dark; *black filled circles*, leaf 1, 10 ms light/dark; *red triangles*, leaf 2, 5 ms light/dark; *blue squares*, leaf 3, 5 ms light/dark. Maximal charge flux and CO_2_ uptake signals were normalized

Simultaneous measurements of CO_2_ uptake and P515 indicated charge flux as a function of CO_2_ concentration were carried out in the presence of 2.1 and 21 % O_2_ using a close to saturating light intensity of 1,120 μmol m^−2^ s^−1^. As shown in Fig. 9a, at 2.1 % O_2_ the shapes of the two CO_2_ response curves are quite similar, when the peak values around 300 μmol mol^−1^ are normalized. The largest relative deviations were found at very low CO_2_ concentrations. They were strongly enhanced when the oxygen concentration was 21 % instead of 2.1 % O_2_, which can be explained by enhanced photorespiration. The ratio of oxygenation to carboxylation increases with decreasing CO_2_ concentration. However, also stimulation of the Mehler-ascorbate peroxidase cycle (MAP cycle) may be involved.

Figure 9b summarizes the relationship between the rates of CO_2_ uptake and charge flux in the presence of 2.1 % O_2_ as a function of CO_2_ concentration as derived from three independent measurements using different leaves and in one case also a different modulation frequency of actinic light (light/dark periods of 10 ms instead of 5 ms). While at high CO_2_ the relationship is close to linear, it becomes curvi-linear at lower CO_2_, with CO_2_ uptake distinctly declining relative to P515 indicated charge flux. This finding agrees with the notion that alternative types of electron transport, like the MAP-cycle (Schreiber and Neubauer 1990; Schreiber et al. 1995), also called water–water cycle (Asada 1999; Miyake 2010), or cyclic PS I (Heber and Walker 1992; Joliot and Joliot 2002, 2005; Joliot and Johnson 2011) are stimulated when electron flow to CO_2_ becomes limited by lack of CO_2_. However, in spite of the low O_2_ concentration present in the experiments of Fig. 9b, also some stimulation of oxygenation (photorespiration) may occur at low CO_2_ concentration.

### Simultaneously measured oscillations of CO_2_ uptake, P515, and charge flux

**Fig. 10:**
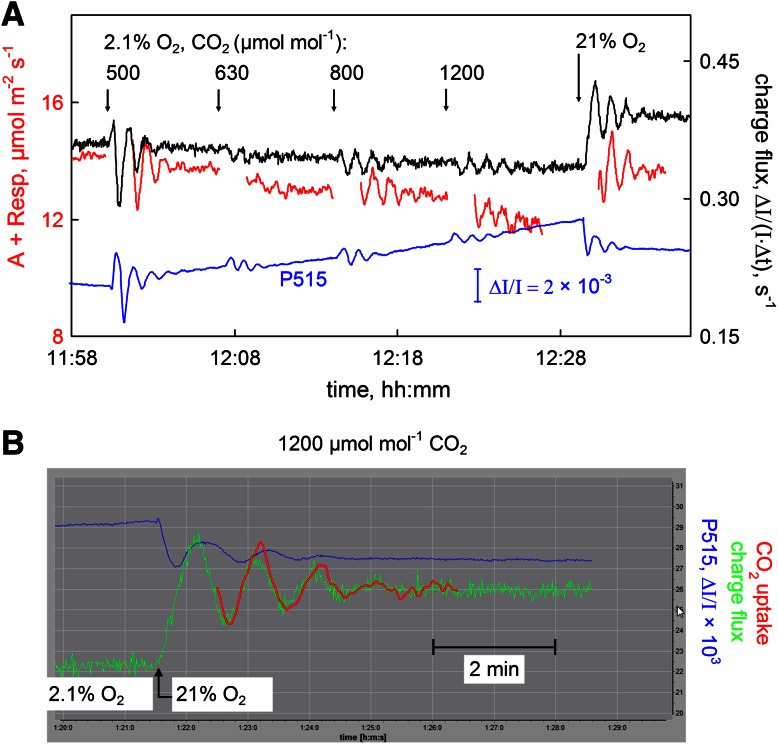
Simultaneous recordings of CO_2_ uptake (*red*), P515 (*blue*), and P515 indicated charge flux (*black*) during regulatory oscillations induced by stepwise increases of CO_2_ concentration from 380 to 500, 630, 800, and 1,200 μmol mol^−1^ and finally by an increase of O_2_ concentration from 2.1 to 21 %. Light intensity, 1120 μmol m^−2 ^s^−1^. Attached dandelion leaf. 10 ms light/dark intervals. **a** Original recordings. **b** Detail of measurement displayed in **a**, based on original screenshot. Oscillations of CO_2_ uptake (*red*), *P515* (*blue*), and P515 indicated charge flux (*green*) induced by a sudden increase of O_2_ concentration from 2.1 to 21 %

Oscillations in photosynthetic parameters have been demonstrated in numerous previous studies and have been discussed in terms of largely differing mechanisms (Sivak and Walker 1986; Furbank and Foyer 1986; Peterson et al. 1988; Stitt and Schreiber 1988; Laisk et al. 1991, 1992; Siebke and Weis 1995; Joet et al. 2001; Nedbal and Brezina 2002). As regulatory oscillations can be observed best in intact leaves, investigations aiming at unraveling their mechanism have been relying primarily on non-invasive indicator signals like Chl fluorescence, light scattering and P700 absorbance at 810–830 nm, measured simultaneously with O_2_ evolution or CO_2_ uptake. In the discussion of the obtained data, apparent phase shifts between the various signals have played a central role. Damped oscillations in CO_2_ uptake can be induced by sudden increases of CO_2_ or O_2_ concentration. Simultaneous measurements of such oscillations in CO_2_ uptake, P515 and P515 indicated charge flux are presented in Fig. 10.

Figure 10a shows the changes in the presence of 2.1 % O_2_ induced by stepwise increases of CO_2_ concentration from 380 to 500, 630, 800, and 1,200 μmol mol^−1^. At the end of the recording 2.1 % O_2_ was replaced by 21 % O_2_. The leaf previously had been illuminated for more than 1 h at close to saturating PAR (1,120 μmol m^−2 ^s^−1^). With every upward jump of CO_2_ concentration and also upon the final increase in O_2_, in all three measured parameters damped oscillations with a period of about 60 s were observed. In Fig. 10b the O_2_-jump response of P515 and charge flux signals is depicted in form of a zoomed screenshot, with the normalized CO_2_ uptake signal on top. A 10 s delay time in the response of the gas analyzer (mainly due to transport of the gas from the cuvette to the analyzer) was taken into account. This delay was determined by injection of microliter amounts of CO_2_ into the cuvette (data not shown). The oscillations in CO_2_ uptake and charge flux are almost synchronous, with the flux signal preceding the uptake signal by not more than 4 s. On the other hand, a significant phase shift of 10–15 s is apparent between these two signals and the P515 signal, with the latter being relatively delayed. The delay between P515 and charge flux signal is of particular analytical value, as the two signals are based on the same measurement and therefore phase shifts due to experimental errors can be excluded.

The data in Fig. 10 show impressively the close relationship between ECS-indicated proton-motive charge flux and CO_2_ uptake, thus confirming the notion that the flux signal provides a close proxy of the rate of photosynthetic electron transport and, hence, may serve as a convenient alternative optical tool for non-invasive in vivo assessment of photosynthesis.

## Summary and conclusions

We have shown that the new dual-wavelength 550–520 nm (P515) module of the Dual-PAM-100 measuring system not only allows to carry out standard DIRK_ECS_ measurements, as extensively described by Kramer and co-workers (reviewed in Kramer et al. 2003, 2004a, b; Avenson et al. 2005a; Cruz et al. 2005), but also provides a new *continuous flux signal*, with which the rate of pmf generation via photochemical charge separation (*R*_ph_) is measured directly and non-invasively. In an example of application of the standard DIRK_ECS_ approach (Fig. 2), we confirmed that partitioning of the overall pmf into ΔpH and Δ*Ψ* components in vivo displays a high extent of flexibility (Cruz et al. 2001; Avenson et al. 2004b). While Δ*Ψ* contributes appreciably at moderately high quantum flux densities, it declines when approaching light saturation, being replaced by ΔpH.

The new *continuous flux* approach (Fig. 4) was conceived to monitor the initial rate of ECS decay during repetitive ms dark-intervals under steady-state as well as changing ECS conditions. Therefore, this new probe can also be used in the investigation of charge fluxes during dark-light induction of photosynthesis, which have played an important role in Pierre Joliot’s recent work on the role of cyclic PS I (CEF1) (reviewed in Joliot and Joliot 2006, 2008; Joliot et al. 2006). We have shown that the new *continuous flux* signal provides practically identical information during dark-light induction as point by point assessment of the initial slopes of ECS decays in particular dark-intervals defined along an induction curve of ECS (Fig. 7). Major advantages of the new probe are the continuity of signal monitoring and the ease of operation. Using the double-modulation approach, with microprocessor controlled signal processing, ambiguities in the assessment of initial slopes are eliminated. Hence, this approach can be even applied reliably by non-experts in absorbance spectroscopy.

We have demonstrated that both the original P515 (ECS) signal and the P515 indicated continuous flux signal (“P515 flux”) can be measured simultaneously with gas exchange (Figs. 8, 9, 10) using a special cuvette developed for parallel measurements of CO_2_ uptake with the GFS-3000 and optical changes (chlorophyll fluorescence, P700, ECS, etc.) with the Dual-PAM-100 and KLAS-100 measuring systems. While in the range of low-to-moderate light intensities the rates of “P515 flux” and CO_2_ uptake were found to be almost linearly correlated, a relative decline of “P515 flux” was observed when saturating light intensities were approached (Fig. 8). It remains to be investigated whether this decline reflects a decrease of H^+^/*e* ^−^ due to saturation of an alternative light-driven pathway that does not involve CO_2_-reduction. This pathway could consist in CEF1 (Heber and Walker 1992; Joliot and Joliot 2006; Laisk et al. 2010), but a participation of the MAP cycle (water–water cycle) may be envisaged as well (Schreiber et al. 1995; Asada 1999; Miyake 2010). At high light intensity and low CO_2_ substantial “P515 flux” was observed that was not paralleled by corresponding CO_2_ uptake (Fig. 9). Again, this finding argues for an alternative, ECS-generating pathway that could be CEF1 or MAP-cycle or both, but at low CO_2_ some contribution of photorespiration cannot be excluded, even at 2.1 % O_2_. Upon sudden increases of CO_2_- or O_2_-concentration, pronounced oscillations in CO_2_ uptake (with period of about 60 s) were found to be paralleled by corresponding oscillations in “P515 flux” and in the original P515 signal (Fig. 10). Interestingly, while oscillations in CO_2_ uptake and P515 flux were almost synchronous, the changes of the original P515 signal were delayed by about 10–15 s with respect to the former two signals.

In this Emerging Techniques report, we were just able to demonstrate a few applications of the new P515 module which allowed a glance on the potential of this new device for advanced studies of various regulatory mechanisms of photosynthetic electron transport. This potential may be considered particularly large, when P515 (ECS) and “P515 flux” are measured simultaneously with other probes of photosynthetic electron transport, like CO_2_-uptake, O_2_-evolution, chlorophyll fluorescence, and P700. After calibration of the flux signal by CO_2_-uptake or O_2_-evolution measurements, it may serve a non-invasive, continuously measured optical proxy of the in vivo rate of photosynthetic electron flow.

## Abbreviations

A: Rate of assimilatory CO_2_ uptake
AL: Actinic light
CEF1: Cyclic electron flow in PS I
cyt bf: Cytochrome b_6_f protein complex
ECS: Electrochromic pigment absorption shift
FR: Far-red light
KLAS: Kinetic LED array spectrophotometer
LED: Light emitting diode
LEF: Linear electron flow
ML: Pulse-modulated measuring light
MAP: Mehler ascorbate peroxidase cycle
NPQ: Non-photochemical quenching
PAM: Pulse amplitude modulation
PAR: Photosynthetically active radiation
P515: Dual-wavelength (550–520 nm) difference signal synonymous with ECS
P700: Dual-wavelength (870–820 nm) difference signal reflecting oxidized P700
P_i_: Inorganic phosphate
pmf: Proton motive force
ΔpH: Proton gradient component of pmf
*R*esp: Rate of day-respiration
*R*_dark_: Rate of dark-interval decay of ECS
*R*_light_: Rate of overall formation of ECS
*R*_bf_: Rate of Q-cycle coupled to cyt bf turnover
*R*_efflux_: Rate of proton efflux via ATP-ase
*R*_ph_: Overall rate of photochemical charge separation
Δ*Ψ*: Electrical component of pmf
SP: Saturation Pulse
ST: Single turnover light pulse
